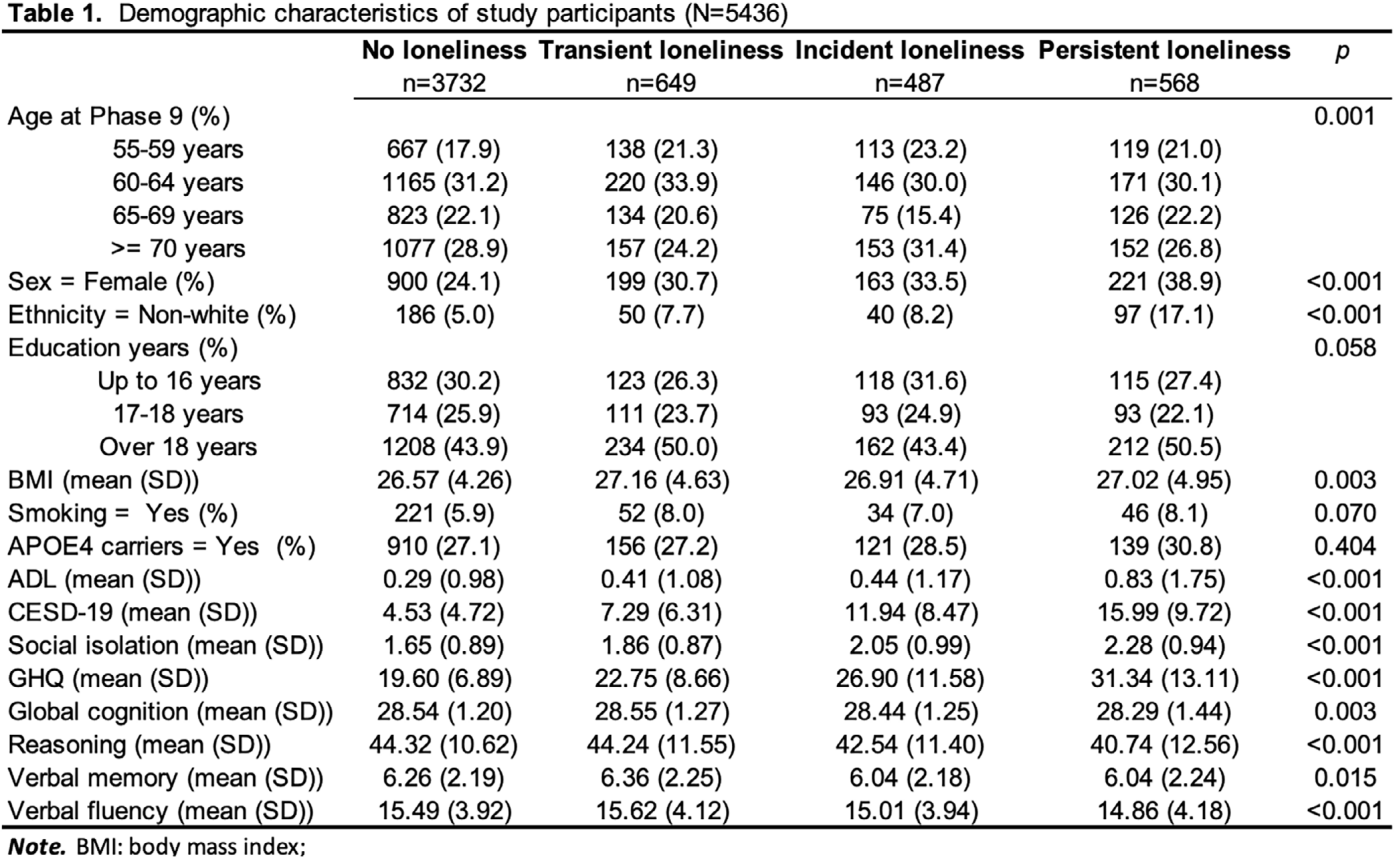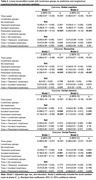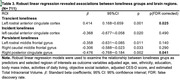# Longitudinal loneliness changes are associated with cognitive decline and brain structure alterations: evidence from the Whitehall II cohort study

**DOI:** 10.1002/alz.084884

**Published:** 2025-01-09

**Authors:** Ye Zhang, Yasuko Tatewaki, Yingxu Liu, Naoki Tomita, Benjamin Thyreau, Yasuyuki Taki

**Affiliations:** ^1^ Tohoku university, Sendai city Japan; ^2^ Tohoku University, Sendai, Miyagi Japan; ^3^ Tohoku University, Sendai Japan; ^4^ Tohoku University Hospital, Sendai Japan

## Abstract

**Background:**

Loneliness has been linked to cognitive decline and an elevated risk of Alzheimer’s disease (AD). Previous studies measured loneliness at a single point time, which may not accurately capture the longitudinal changes of different loneliness types (e.g., recover form loneliness; persistent loneliness) and their impact in the neuropathology of AD. This study investigates five‐year longitudinal changes of loneliness and their associations with cognitive function and brain structure alterations using data from the Whitehall II cohort study.

**Method:**

This study included 5346 participants with mean age = 65 year (Female:26.68%) at baseline (2007‐2009). Loneliness was measured using one item form the Center for Epidemiologic Studies Depression Scale, and longitudinal loneliness changes were defined from two time points (2002‐2004; 2007‐2009). Linear mixed‐effect models were used to examine the association between different loneliness types and eight‐year changes of different cognitive domains (global cognition, reasoning, verbal fluency, and verbal memory). Robust linear regression models were employed to assess loneliness types and gray matter volumes (GMVs) in selected brain regions five‐year later (2012‐2012) using the Whitehall IIMRI substudy (N = 711).

**Result:**

We defined four types of loneliness changes: “No loneliness”, “Transient loneliness”, “Incident loneliness” and “Persistent loneliness”. Compared to no loneliness, persistent loneliness was associated with decline in cognitive function (global cognition: b = ‐0.20, p<0.001; reasoning: b = ‐0.29, p<0.001; verbal memory: b = ‐0.11, p<0.007; verbal fluency: b = ‐0.15, p = 0.001;) after controlled for age, sex, education, and objective social isolation. Compared to no loneliness, persistent loneliness was associated with decreased GMVs in right posterior cingulate cortex (b = ‐0.17, p_FDR‐corrected_ = 0.041); transient loneliness was associated with increased GMVs in rostral anterior cingulate cortex (b = ‐0.48, p_FDR‐corrected_ = 0.025).

**Conclusion:**

Persistent loneliness was associated with both longitudinal cognitive decline and decreased brain regions involved in emotional regulation, suggesting persistent loneliness feelings, no incident loneliness, could be a risk factor in the neuropathology of AD. Moreover, recovery from loneliness feeling (transient loneliness) could be protective in future brain structure alterations. Our study suggests that identifying participants has long‐term loneliness and developing strategies to overcome loneliness feeling could be important for AD prevention.